# Two-dimensional organic-inorganic molecular cocrystal

**DOI:** 10.1093/nsr/nwaf476

**Published:** 2025-11-08

**Authors:** Yiran Ma, Zhichen Xu, Haidi Liu, Xin Feng, Jie Liu, Decai Ouyang, Zongdong Sun, Huihui Ding, Lingyi Ao, Yue Hu, Hao Peng, Dehui Li, Yingshuang Fu, Hongtao Yuan, Yongli Yan, Yuanping Yi, Meihui Wang, Tianyou Zhai

**Affiliations:** State Key Laboratory of Materials Processing and Die & Mould Technology, School of Materials Science and Engineering, Huazhong University of Science and Technology (HUST), Wuhan 430074, China; Beijing National Laboratory for Molecular Sciences, CAS Key Laboratory of Organic Solids, Institute of Chemistry, Chinese Academy of Sciences, Beijing 100190, China; Key Laboratory of Photochemistry, Institute of Chemistry, Chinese Academy of Sciences, Beijing 100190, China; State Key Laboratory of Materials Processing and Die & Mould Technology, School of Materials Science and Engineering, Huazhong University of Science and Technology (HUST), Wuhan 430074, China; State Key Laboratory of Materials Processing and Die & Mould Technology, School of Materials Science and Engineering, Huazhong University of Science and Technology (HUST), Wuhan 430074, China; State Key Laboratory of Materials Processing and Die & Mould Technology, School of Materials Science and Engineering, Huazhong University of Science and Technology (HUST), Wuhan 430074, China; State Key Laboratory of Materials Processing and Die & Mould Technology, School of Materials Science and Engineering, Huazhong University of Science and Technology (HUST), Wuhan 430074, China; National Laboratory of Solid State Microstructures, College of Engineering and Applied Sciences, and Collaborative Innovation Center of Advanced Microstructures, Nanjing University, Nanjing 210093, China; National Laboratory of Solid State Microstructures, College of Engineering and Applied Sciences, and Collaborative Innovation Center of Advanced Microstructures, Nanjing University, Nanjing 210093, China; School of Optical and Electronic Information and Wuhan National Laboratory for Optoelectronics, Huazhong University of Science and Technology, Wuhan 430074, China; State Key Laboratory of Materials Processing and Die & Mould Technology, School of Materials Science and Engineering, Huazhong University of Science and Technology (HUST), Wuhan 430074, China; School of Optical and Electronic Information and Wuhan National Laboratory for Optoelectronics, Huazhong University of Science and Technology, Wuhan 430074, China; School of Physics, Huazhong University of Science and Technology, Wuhan 430074, China; National Laboratory of Solid State Microstructures, College of Engineering and Applied Sciences, and Collaborative Innovation Center of Advanced Microstructures, Nanjing University, Nanjing 210093, China; Jiangsu Physical Science Research Center, Nanjing 210093, China; Key Laboratory of Photochemistry, Institute of Chemistry, Chinese Academy of Sciences, Beijing 100190, China; Beijing National Laboratory for Molecular Sciences, CAS Key Laboratory of Organic Solids, Institute of Chemistry, Chinese Academy of Sciences, Beijing 100190, China; State Key Laboratory of Materials Processing and Die & Mould Technology, School of Materials Science and Engineering, Huazhong University of Science and Technology (HUST), Wuhan 430074, China; State Key Laboratory of Materials Processing and Die & Mould Technology, School of Materials Science and Engineering, Huazhong University of Science and Technology (HUST), Wuhan 430074, China; Research Institute of Huazhong University of Science and Technology in Shenzhen, Shenzhen 518057, China

**Keywords:** 2D molecular cocrystal, intermolecular interaction, photoluminescence, optical waveguide

## Abstract

Molecular cocrystal engineering has emerged as an efficient approach to achieving multiple functionalities and novel applications in photonics and optoelectronics. However, the reported two-dimensional (2D) molecular cocrystals are predominantly composed of organic molecules, whereas the preparation of organic-inorganic molecular cocrystals (OIMCs) still remains challenging. Here, we achieved the self-assembly of the 2D OIMC C_60_·2P_4_S_3_ for the first time, which has a stable layered packing arrangement, driven by π-π interactions and C-P contacts between these two cage molecules. We found that the strong C-P contacts suppress non-radiative transitions and enhance vibrational coupling, resulting in an efficient emission with a quantum yield of 13.24%. Unlike the molecular units of C_60_ and P_4_S_3_, the cocrystal demonstrates asymmetric optical waveguides with a high anisotropic ratio of 3.625, which can be attributed to the anisotropy of vibrational transition. This work can provide new light on the design of molecular cocrystals and promote the development of molecular physics and optoelectronics.

## INTRODUCTION

Two-dimensional (2D) molecular cocrystals, formed by two or more molecules held together through intermolecular interactions, have attracted significant interest for their multifunctional properties and promising applications in photonics and optoelectronics [1–3]. And the synergistic effects between different molecular building blocks often lead to unique optical properties that individual components do not possess, offering a valuable platform for studying novel phenomena [4,5]. Based on a comprehensive materials database, researchers have reported various cocrystals with tunable carrier transport and optical properties [6–8]. However, most reported 2D molecular cocrystals are solely composed of organic molecules, leaving a blank in the development of 2D organic-inorganic molecular cocrystals (OIMCs). While such hybridization holds great promise for enhancing optical properties, exploration of 2D OIMCs has been limited by distinct intermolecular interactions within organic or inorganic molecules [9,10]. Specifically, organic molecules usually exhibit interactions such as π-π interaction and hydrogen bonding, while inorganic molecules form various close intermolecular contacts [1,11–13]. The understanding of the complex force fields governing interactions in organic-inorganic molecular systems remains limited.

Herein, we report a 2D OIMC C_60_·2P_4_S_3_ for the first time, whose conjugated cage-like frameworks promote ordered packing through molecular recognition [14–20]. With the strong interaction between C_60_ and P_4_S_3_, this cocrystal exhibits efficient photoluminescence and excellent anisotropic optical waveguide performance. C_60_·2P_4_S_3_ demonstrates atomically smooth 2D surfaces, nanometer-scale thicknesses, and high crystallinity. This cocrystal consists of alternating layers of C_60_ and P_4_S_3_ with short C-P contacts between the layers, which was identified as a key factor driving the self-assembly process. We found that this network of C-P contacts effectively suppresses non-radiative transitions and enhances vibronic coupling transition. As a result, C_60_·2P_4_S_3_ nanoflakes demonstrate efficient optical emission with a photoluminescence quantum yield (PLQY) of 13.24%, which represents the highest value reported to date for C_60_-based luminescent materials. Due to the anisotropy of the transition dipole moment caused by vibration coupling, this C_60_·2P_4_S_3_ crystal presents an asymmetric optical waveguide with an anisotropic ratio of 3.625.

## RESULTS AND DISCUSSION

**Figure 1.**
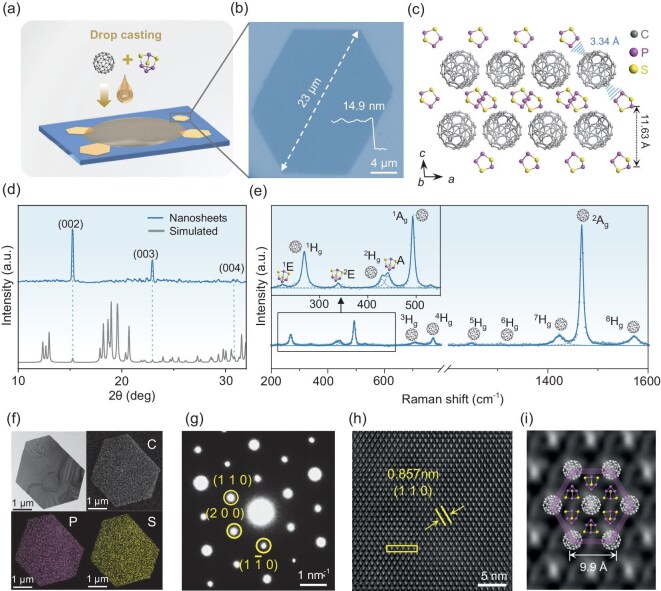
Preparation and characterization of 2D C_60_·2P_4_S_3_ cocrystals. (a) Schematic of the preparation process for 2D C_60_·2P_4_S_3_ cocrystals by a drop-casting method. (b) Optical image of the as-grown C_60_·2P_4_S_3_ nanoflake and the corresponding height profile measured by atomic force microscope. (c) Crystal structure of C_60_·2P_4_S_3_ along the *b*-axis direction. The blue bars represent the short contacts between C_60_ and P_4_S_3_ molecules. (d) XRD pattern of the as-grown C_60_·2P_4_S_3_ nanoflakes. (e) Raman spectrum of the 2D C_60_·2P_4_S_3_. (f) TEM image of a C_60_·2P_4_S_3_ nanoflake and the corresponding elemental maps for C, P, and S. (g) SAED pattern of the C_60_·2P_4_S_3_ nanoflake. (h) IFFT image corresponding to the HRTEM image of the nanoflake (Fig. S3b). (i) Enlarged IFFT image and the matched crystal structure with (001) plane.

As shown in Fig. 1a, 2D C_60_·2P_4_S_3_ cocrystals were prepared by a simple drop-casting method. Specifically, 2–3 µl of a mixed solution of C_60_ and P_4_S_3_ (0.10 wt% C_60_ and 0.08 wt% P_4_S_3_ dissolved in toluene), was dropped onto a SiO_2_/Si wafer. As the supersaturated solution gradually evaporated at room temperature, the C_60_ and P_4_S_3_ molecules co-assembled into hexagonal nanoflakes with thicknesses ranging from 7.98 nm to 100 nm and lateral areas varying from 5 µm to 60 µm (Fig. S1). The well-defined hexagonal shapes of these nanoflakes reflect their high degree of crystallinity and good stability (Fig. S2).

Single-crystal X-ray diffraction (SCXRD) analysis confirmed that the as-grown nanoflakes are C_60_·2P_4_S_3_ cocrystals, which crystallize in the monoclinic *C*2/*m* space group with lattice parameters of *a* = 17.4098 Å, *b* = 9.8283 Å, *c* = 11.6240 Å (Table S1). Figure 1c demonstrates the crystal structure of C_60_·2P_4_S_3_ along the *b*-axis direction, showing the segregated stacking structure with alternating layers of C_60_ and P_4_S_3_ along the *c*-axis direction. Within the C_60_ layers, each fullerene molecule adopts a quasi-hexagonal pattern, surrounded by six nearest neighbors at an intermolecular distance of ∼3.26 Å, giving rise to a periodic nanocluster topology (Fig. S3) [18]. An interlayer spacing between two adjacent P_4_S_3_ layers was measured to be 11.63 Å. The X-ray diffraction (XRD) pattern of the as-grown C_60_·2P_4_S_3_ nanoflakes on the SiO_2_/Si wafer shows three sharp diffraction peaks, which match well with the (002), (003), and (004) planes of the simulated patterns (Fig. 1d), indicating the exposed (top) surface of the as-grown cocrystals is the (001) facet. Figure 1e presents the Raman spectrum of a 2D C_60_·2P_4_S_3_ cocrystal using a 532 nm excitation laser. A total of 14 Raman modes were identified, including 3 modes (2E + A) attributed to P_4_S_3_, 10 modes (2A_g_ + 8H_g_) assigned to C_60_, and an unidentified peak at 530 cm^−1^ [21,22]. Compared with bulk P_4_S_3_, the cocrystal shows a ^2^E mode blueshifting from 338 to 342 cm^−1^. Conversely, vibrational modes of C_60_ exhibited a redshift: ^1^H_g_ shifted from 272 to 269 cm^−1, 1^A_g_ from 496 to 494 cm^−1^, and ^5^H_g_ from 710 to 708 cm^−1^. These shifts likely result from the van der Waals intermolecular interactions and symmetry breaking caused by the presence of inorganic molecules [23–26]. Notably, the ^2^A_g_ mode of C_60_, which is highly sensitive to charge transfer, remained unchanged at 1468 cm^−1^. This indicates no significant charge transfer occurring within the cocrystal [23].

The chemical composition and crystal structure of the obtained C_60_·2P_4_S_3_ cocrystal were further investigated using a transmission electron microscope (TEM). The C, P, and S elemental maps (Fig. 1f and Fig. S4a) show a uniform distribution over the whole hexagonal nanoflake. The selected area electron diffraction (SAED) pattern exhibits sharp spots with one orientation, indicating the high crystallinity of the as-grown nanoflake and well matched to the (001) top plane of the C_60_·2P_4_S_3_ cocrystal (Fig. 1g). High-resolution TEM (HRTEM) image (Fig. S4b) and the corresponding inverse fast Fourier transform (IFFT) images (Fig. 1h) revealed a hexagonal lattice with a spacing *d* = 0.857 nm, corresponding to the (110) plane of C_60_·2P_4_S_3_. The lattice structure is further verified by the intensity line profile along the yellow box in Fig. 1h. The periodic intensity curve (Fig. S4c) highlights strong peaks corresponding to the C_60_ cage molecules, which are heavier and exhibit stronger contrast. Figure 1i shows the enlarged IFFT image along with the simulated crystal structure from SCXRD (Fig. S3c). The alternating light and shaded regions in the (001) plane correspond to C_60_ and P_4_S_3_ molecules, respectively.

**Figure 2.**
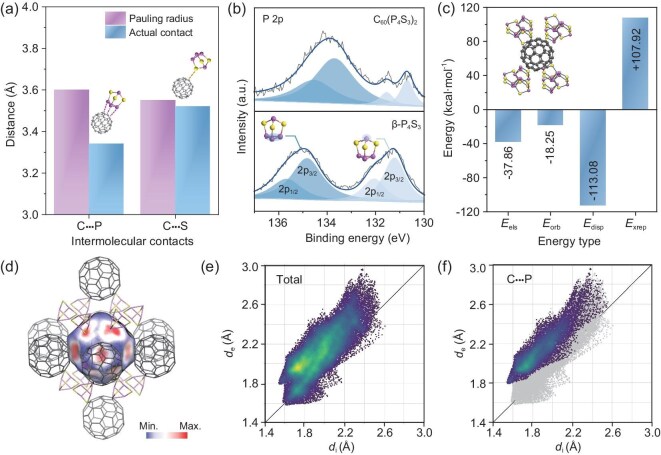
Intermolecular interactions in 2D C_60_·2P_4_S_3_ cocrystals. (a) Short intermolecular contacts observed in C_60_·2P_4_S_3_. (b) XPS P 2p spectra of C_60_·2P_4_S_3_ and β-P_4_S_3_. (c) Energy decomposition analysis of C_60_·2P_4_S_3_. The electrostatic term (*E*_els_), the orbital term (*E*_orb_), the dispersion term (*E*_disp_), and the exchange term (*E*_xrep_) encompass electrostatics, induction, dispersion force, and exchange interaction, respectively. (d) Hirshfeld surface of the C_60_ molecule in the 2D C_60_·2P_4_S_3_ cocrystal. (e) 2D fingerprint plots of the C_60_ molecule shown in Fig. 2d, depicting the distance between the Hirshfeld surface and the nearest interior atom (*d*_i_), and the nearest exterior atom surface (*d*_e_). (f) Local contact fingerprint plots between the C_60_ molecule and neighboring P atoms.

To investigate the co-assembly behaviors of the organic molecule C_60_ and the inorganic molecule P_4_S_3_, a comprehensive analysis of the intermolecular interactions was performed by both experimental characterizations and theoretical calculations. SCXRD results reveal an unusually short C-P contact between the C_60_ and P_4_S_3_ molecules (Fig. 1c). Among the neighboring P_4_S_3_ molecules around the central C_60_ molecule, the P atoms located on the ternary ring at the bottom of P_4_S_3_ cages are the closest to the C_60_. The observed C-P contact distance (blue bars in Fig. 1c) is 3.34 Å, which is shorter than the theoretical sum of the Pauling–van der Waals radii (3.60 Å), whereas the minimum C-S contact (3.52 Å) closely matches the theoretical value of 3.55 Å (Fig. 2a) [27]. The unusually short C-P contacts observed in the nanoflakes suggest strong intermolecular interactions, which further influence the electron cloud distribution between the molecules. As shown in Fig. 2b, the XPS P 2p spectrum of the cocrystal shows two distinct chemical states for the P atoms, which were further fitted into four peaks (134.54 eV, 133.70 eV, 131.54 eV, and 130.70 eV). It was reported that the two peaks with the higher binding energy correspond to the P atoms on the bottom ternary ring (dark blue), while the peak with the lower binding energy represents the top P atom (light blue) [28]. Compared to β-P_4_S_3_, the P atoms in the cocrystals exhibit lower binding energies, where the bottom P shows a particularly large shift of 1.13 eV. The reduction in binding energy in the nanoflakes can be directly linked to the unusually short C-P contacts, which enable electron sharing between the C_60_ molecules and the nearest-neighboring P atoms, thereby increasing the electron density around the P atoms [11]. The binding energy of S 2p remains nearly unchanged (Fig. S5a), which is consistent with the C-S contact distance observed in SCXRD. The broadening of the C 1s peak (Fig. S5b) also indicates that the strong intermolecular C-P contacts in the cocrystal alter the local electronic environment of the C atoms. A detailed comparison is provided in Table S2. In energy decomposition analysis (Fig. 2c), the strong C-P contacts give rise to a substantial dispersion interaction (−113.08 kcal·mol^−1^), while the electronegativity difference between the two atoms contribute to the electrostatic and induction terms. It is noteworthy that the exchange term exhibits a large contribution, with a value of +107.92 kcal·mol^−1^. This may be attributed to the considerable wave function overlap in the cocrystal, which has been reported in certain organic materials [29]. Considering the π-character of electrons on both C_60_ and the basal P_3_ ring of P_4_S_3_, it is suggested that π-π interactions also exist between them [5,20].

We further simulated the Hirshfeld surface diagram of the C_60_ molecule in the 2D C_60_·2P_4_S_3_ cocrystals, colored by electron density (Fig. 2d and Fig. S6). In this diagram, red indicates stronger interactions, while blue corresponds to weaker interactions. It was observed that the C_60_ molecule has strong intermolecular coupling with the surrounding C_60_ molecules (intralayer) and P atoms (interlayer). Remarkably, strong intermolecular interactions are observed between the C_60_ molecule and the bottom P atoms on the ternary ring of the P_4_S_3_ cage, thereby validating the strong C-P contact. As revealed by the fingerprint diagram (Fig. 2e) and local contact analysis (Fig. 2f), the characteristic ‘spike’ region of C-P contact is located where *d*_i_ < *d*_e_ (*d*_i_ = 1.55 Å, *d*_e_ = 1.79 Å). This suggests that the surface is closer to the C atom, indicating a higher charge density near the P atom. In this context, the P atom behaves similarly to a hydrogen bond donor in systems involving hydrogen bonding, where the H atom donates electrons to the more electronegative atom (hydrogen donor), resulting in the surface being closer to the H atom [30]. Thus, we propose that a strong C-P contact contributes to the local charge redistribution. Generally, the segregated packing of C_60_·2P_4_S_3_ cocrystals originates from the synergetic effect of both the intensive π-π interactions between C_60_ cages and the network of strong C-P contact.

**Figure 3.**
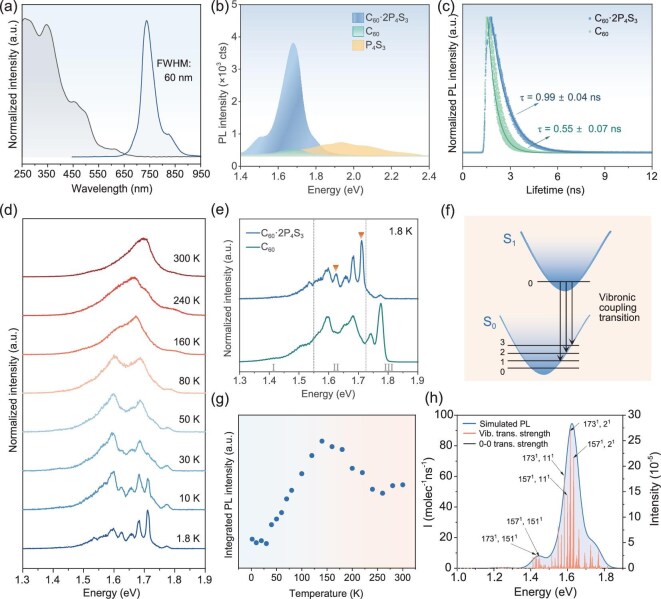
Photophysical characterization of 2D C_60_·2P_4_S_3_ cocrystals. (a) Absorption and PL spectra of the cocrystals. (b) PL spectra for C_60_·2P_4_S_3_, C_60_ nanoflakes and P_4_S_3_ particles. (c) PL decay profiles for C_60_·2P_4_S_3_ and C_60_ nanoflakes. (d) Temperature-dependent PL spectra ranging from 1.8 to 300 K. (e) PL spectra at 1.8 K of C_60_·2P_4_S_3_ cocrystal and C_60_. (f) Schematic of vibronic coupling transition. (g) Temperature dependence of PL intensity from 1.8 to 300 K. (h) Vibronic assignments for the dominant peaks of one photon emission spectrum.

As shown in Fig. 3a, the photoluminescence (PL) spectrum of the cocrystals exhibits a broad full width at half maximum (FWHM) of 60 nm. The PL intensity gradually increases with thickness maintaining nearly unchanged peak positions (Fig. S7a and b) and follows a power-law dependence on the excitation power, with a power-law exponent (*k*) of 0.96 ± 0.09 (Fig. S7c and d). A *k* value close to 1 indicates that recombination of photogenerated carriers is mainly governed by localized excitons, which is consistent with previous reports [31]. Furthermore, the emission peak position of the cocrystal coincides with that of pristine C_60_ (Fig. S7e). Together with absorption spectra and band structure calculations (Figs S7f, S8, and S9), these results confirm the absence of electron transfer occurring between the two components, indicating that the luminescent center of the cocrystal originates from C_60._

Notably, a substantial enhancement in PL intensity is observed compared with C_60_ (Fig. 3b). The PLQY of C_60_·2P_4_S reaches 13.24% at room temperature, two orders of magnitude higher than that of pristine C_60_ (0.07%), where emission is typically quenched by dipole-forbidden singlet exciton recombination [32]. This PLQY is also superior to most fullerene-based materials reported to date (Table S3), demonstrating that the environment in the cocrystal strongly promotes C_60_ emission. Besides, time-resolved PL measurements further support this conclusion. As shown in Fig. 3c and Fig. S10, the average lifetime of the cocrystal is 0.99 ± 0.04 ns, which is longer than that of the C_60_ nanoflake (0.55 ± 0.07 ns). The extended lifetime confirms that the molecular cocrystal exhibits a longer-lived excited state than the C_60_ nanoflake, indicating reduced non-radiative relaxation processes such as internal conversion and intermolecular energy transfer. This behavior can be attributed to the effective shielding of C_60_ molecules by the rigid inorganic P_4_S_3_ units, together with the compact molecular packing stabilized by strong C-P contacts [33].

To further investigate its optical transitions, the PL spectra of the cocrystal were measured over a temperature range of 1.8–300 K. As shown in Fig. 3d, the PL peak gradually splits into two distinguishable peaks at ∼1.60 eV and 1.68 eV as the temperature decreases from 300 to 80 K. As the temperature continues to drop, the thermal motion of molecules slows down, and the quantum effects become pronounced, leading to the well-developed fine structure of the vibrational energy level, which corresponds to Herzberg–Teller-induced vibronic transitions [34]. This vibronic transition is characteristic of localized exciton recombination, which correlates well with the previously discussed *k* value as being close to 1 [31]. The distinct peaks in the spectra represent the excitation of different intramolecular vibrations of C_60_ in C_60_·2P_4_S_3_ nanoflakes. The energies of the vibronic bands relative to the origins for 2D C_60_·2P_4_S_3_ are summarized in Table S4 [35].

The low-temperature PL spectra are reportedly divided into three spectral regions associated with distinct radiative recombination processes in different energy (*E*) intervals: region Ⅰ (*E* ≤ 1.55 eV), region Ⅱ (1.55 eV ≤ *E* ≤ 1.72 eV), and region Ⅲ (*E* ≥ 1.72 eV) [34,36]. Region Ⅰ corresponds to the electronic-vibrational transition of the Frenkel exciton from the first excited triplet state to the ground state (S_0_), region Ⅱ represents the transition from the first excited singlet state (S_1_) to S_0_, and region Ⅲ corresponds to the radiative recombination from X-traps. In other C_60_-based crystals, the intermolecular interactions are generally weak compared to the strong intra-molecular interactions within the C_60_ molecule itself, thus having little effect on the vibronic frequencies of C_60_ [37]. However, comparison of the fine structures of C_60_·2P_4_S_3_ and C_60_ at 1.8 K reveals that this cocrystal system exhibits a more complex vibrational fine structure including peaks at 1.625 eV and 1.712 eV, which is a result of the enhanced interaction between the constituent components at lower temperatures (Fig. 3e). Notably, the vibrational energy level at 1.712 eV in C_60_·2P_4_S_3_, which is likely associated with an unidentified Raman mode observed at 530 cm^−1^, demonstrates the highest emission intensity. This contrasts markedly with pristine C_60_, where the dominant emission originates from X-traps. This suggests that the strong intermolecular contacts in C_60_·2P_4_S_3_ strengthens vibronic coupling, leading to an increased probability of singlet-state transition (Fig. 3f) [38]. The temperature dependence of the PL intensity is summarized in Fig. 3g. The intensity exhibits a pronounced increase as the temperature rises from 1.8 K, reaching a maximum at ∼140 K, followed by a gradual decrease upon further heating. This is probably because the C_60_ molecules transition from a completely rotationally locked state to a state of motion with increasing temperature. This transition facilitates the population of high-energy vibrational modes, which in turn strengthens the vibrationally coupled emission, leading to the observed increase in PL intensity. However, as the temperature continues to rise above 140 K, the PL intensity undergoes a decline, primarily due to the onset of thermal quenching effects, which dominate at elevated temperatures [39]. To gain a deeper understanding of the vibronic coupling accompanying the optical transition, theoretical calculations of the vibrationally-resolved electronic spectra were conducted (Fig. 3h and Fig. S11). Asymmetry between the emission and absorption spectra is caused by the change in geometrical structure and vibrations with Herzberg–Teller couplings [40]. It is observed that the 0–0 transition is almost negligible, while multiple vibrational transitions exhibit high intensity. This implies that the oscillator strength for electronic transitions in the equilibrium configuration vanishes in the absence of vibrations, but during vibrational excitation, the geometry temporarily deviates from equilibrium, resulting in a non-zero transition dipole moment and oscillator strength for the electronic transitions. Figure 3h and Table S5 present the vibronic assignments for the major peaks in the emission spectra, revealing that the emission primarily originates from dual-mode vibronic excitation dominated by high-frequency modes (typically modes 157 or 173) and low-frequency modes (typically modes 11 or 2).

Pressure engineering has recently been utilized on the 2D crystals to study their physical and chemical properties [41,42]. The PL spectra of the C_60_·2P_4_S_3_ nanoflakes under high pressure up to 1.54 GPa were recorded using a symmetrical diamond anvil cell, as shown in Fig. S12. As the pressure increases, a decrease in fluorescence intensity is observed, accompanied by the splitting of the fluorescence peak into two distinct peaks. As mentioned in the simulated emission spectrum, peak A corresponds to the mixture of high-frequency and low-frequency modes, while peak B, at lower energy, is dominated by high-frequency bond stretching. The pressure coefficients for peaks A and B are 36.1 meV·Gpa^−1^ and 73.1 meV·Gpa^−1^, respectively. The disparity in the pressure-induced shifts between these peaks can be attributed to the differential response of vibrational modes to pressure. Specifically, low-frequency vibrations, which are more susceptible to pressure modulation compared to high-frequency vibrations originating from intramolecular bond stretching, dominate the response. The absence of significant changes in the PL spectra before and after applying pressure indicates the cocrystal structure remains intact (Fig. S12d).

To sum up, two factors were found that contribute to the strong emission observed in C_60_·2P_4_S_3_ nanoflakes. First, the incorporation of polar molecules generates an inhomogeneous electrostatic environment around the C_60_ molecule, which modulates the local electron distribution and thereby leads to the enhanced electron-vibronic coupling [43]. In the presence of the Herzberg–Teller effect, efficient optical transition can be generated in C_60_·2P_4_S_3_ cocrystals [38]. Second, the segregated packing of molecules allows the P_4_S_3_ units to arrange in a barrier-like configuration, shielding the adjacent C_60_ molecules from collision and preventing non-radiative decay.

**Figure 4.**
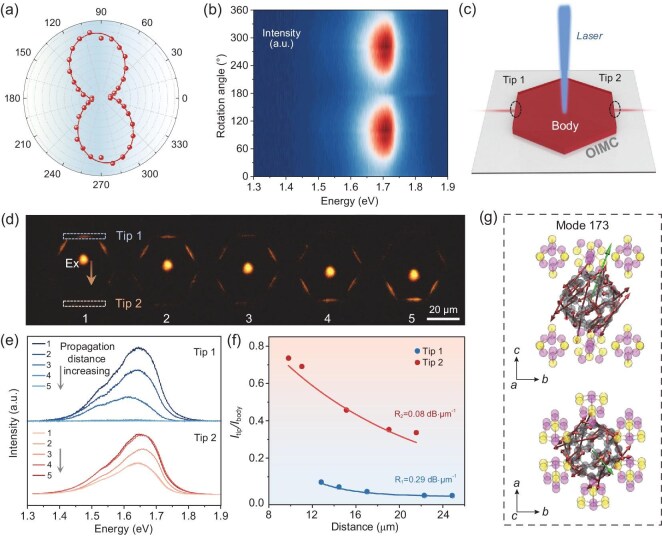
Anisotropic optical properties of 2D C_60_·2P_4_S_3_ cocrystals. (a) Polar plot of the PL intensity. (b) Angle-resolved polarized 2D false-color PL spectra. (c) Illustration of the anisotropic optical waveguide. (d) FM images obtained from an individual C_60_·2P_4_S_3_ cocrystal by exciting with a laser beam (*λ* = 405 nm) at different positions along the [100] direction with a scale bar of 20 µm. (e) The corresponding spatially resolved PL spectra in (e) for the tip 1 (above) and 2 (bottom). (f) The ratios of the intensity *I*_tip_/*I*_body_ against the distance corresponding to (e). Curves were fitted by an exponential decay function *I*_tip_/*I*_body_ = *A*exp(−*RD*), where *A* is the ratio of energy escaping and propagating, and *D* is the distance between the excited site and the emitting edge. (g) Schematic of DTDM from mode 173. The red arrow represents the direction of atomic motion, and the green arrow represents the direction of the DTDM.

Furthermore, we measured the anisotropic optical properties of this 2D OIMC. As shown in Fig. 4a and b, the PL emission of the C_60_·2P_4_S_3_ nanoflake is polarization-dependent with a single axis of anisotropy, while the C_60_ nanoflake displays isotropic emission (Fig. S13). When the collection polarizer is oriented at an angle θ = 100° relative to the (010) direction of the flake, the PL intensity reaches its maximum. The emission dichroic ratio, *R*_d_ = *I*_100_/*I*_10_, is found to be 3.04, corresponding to a polarization ratio *ρ* = 0.51, where *ρ* = (*R*_d_ − 1)/(*R*_d_ + 1).

In addition to its anisotropic PL, the C_60_·2P_4_S_3_ nanoflake, exhibiting a small overlap between emission and absorption along with a high PLQY, shows good potential for optical waveguide applications. By moving a 405 nm laser spot across the cocrystal nanoflake, PL spectra were collected from both ends of the flake, labelled as tip 1 and tip 2, as shown in Fig. 4c. Fluorescence microscopy (FM) images in Fig. 4d indicate the 2D optical waveguide behavior, where both the intensity of bright edges (*I*_tip_) and body (*I*_body_) were recorded. Figure 4e provides the PL intensity recorded at two emitting edges. The ratio of *I*_tip_/*I*_body_ shows a single-exponential decay as a function of propagation distance. Notably, the 2D C_60_·2P_4_S_3_ cocrystal demonstrates asymmetric emission at the two edges (Fig. 4f). For propagation towards tip 1 and tip 2, the optical-loss coefficients (*R*) are *R*_1_ = 0.29 dB·µm^−1^ and *R*_2_ = 0.08 dB·µm^−1^, yielding an anisotropic waveguide ratio *R*_1_/*R*_2_ = 3.625. Compared with other organic molecular cocrystals, C_60_·2P_4_S_3_ has a low optical loss combined with pronounced anisotropy (Fig. S14). The exceptional anisotropic optical waveguide characteristics suggest that 2D cocrystals hold great potential for photonic integrated circuits.

It was reported that the orientation of the optical transition dipole in molecular crystals affects the refractive index, PL polarization state, and light propagation properties [44,45]. In the 2D C_60_·2P_4_S_3_ cocrystal, the emission arises from the Herzberg–Teller effect, so the distinct orientations of vibrational modes move the observed changes in the direction of the transition dipole moment. We propose that under the condition of sufficiently small vibrational displacements, the transition dipole moment induced by the Herzberg–Teller effect can be approximated as the first derivative of the transition dipole moment (DTDM) at the equilibrium position. As shown in Fig. 4g and Fig. S15, the directions of DTDMs for the high-frequency modes 173 and 157 are illustrated by theoretical calculations. The projections of these derivatives along the crystallographic axes are detailed in Table S6, where the larger projections along the *b*-axis compared to the *a*-axis for these high-frequency modes account for the observed in-plane anisotropic optical properties.

## CONCLUSION

In conclusion, a new 2D OIMC C_60_·2P_4_S_3_ was successfully designed and prepared. The strong intermolecular interactions between the organic and inorganic components were revealed through a combination of SCXRD, XPS, and Hirshfeld surface analysis. We demonstrated that the formation of a segregated structure with alternating stacks of C_60_ and P_4_S_3_ molecules is driven by the π-π interactions and short C-P contacts. Remarkably, this 2D OIMC shows efficient fluorescence emission with a PLQY of 13.24%, representing the highest value reported thus far for C_60_-based luminescent materials. The temperature-dependent and simulated PL spectra analysis reveals that the observed intense emission results from two main factors. First, the strong intermolecular interactions lead to significant vibrational coupling. Second, the segregated packing in the 2D OIMC reduces non-radiative transitions. Furthermore, due to the anisotropic nature of the transition dipole moment caused by this vibrational coupling, anisotropic PL emission and asymmetric optical waveguide properties were observed in the C_60_·2P_4_S_3_ nanoflakes, with an anisotropy waveguide ratio of 3.625. Our work provides valuable insights into the role of molecular packing in 2D OIMCs and paves the way for future research in molecular physics and chemistry, as well as the development of advanced applications in photonics and optoelectronics.

## Supplementary Material

nwaf476_Supplemental_File
