# Supplementary material for: Two-dimensional organic-inorganic molecular cocrystal
Source: Natl Sci Rev. 2025 Nov 8;13(2):nwaf476. doi: 10.1093/nsr/nwaf476 (PMC12839525; doi:10.1093/nsr/nwaf476)
Supplement: nwaf476_Supplemental_File [file nwaf476_supplemental_file.pdf]

Supplementary data

Two-dimensional organic-inorganic molecular cocrystal

Yiran Ma,[1,†] Zhichen Xu,[2, †] Haidi Liu,[3] Xin Feng,[1] Jie Liu,[1] Decai Ouyang,[1] Zongdong Sun,[1] Huihui Ding,[4] Lingyi Ao,[4] Yue Hu,[5] Hao Peng,[1] Dehui Li,[5] Yingshuang Fu,[6] Hongtao Yuan,[4] Yongli Yan,[3,]* Yuanping Yi,[2,]* Meihui Wang,[1] and Tianyou Zhai[1,7,]*

1 State Key Laboratory of Materials Processing and Die & Mould Technology, School of Materials Science and Engineering, Huazhong University of Science and Technology (HUST), Wuhan 430074, P. R. China

2 Key Laboratory of Organic Solids, Institute of Chemistry, Beijing National Laboratory for Molecular Sciences, Beijing 100190, P. R. China

3 Key Laboratory of Photochemistry, Institute of Chemistry, Chinese Academy of Sciences, Beijing 100190, P. R. China

4 National Laboratory of Solid State Microstructures, Jiangsu Key Laboratory of Artificial Functional Materials, College of Engineering and Applied Sciences, and Collaborative Innovation Center of Advanced Microstructures, Nanjing University, Nanjing 210000, P. R. China

5 School of Optical and Electronic Information and Wuhan National Laboratory for Optoelectronics, Huazhong University of Science and Technology, Wuhan 430074, P. R. China

6 School of Physics and Wuhan National High Magnetic Field Center, Huazhong University of Science and Technology, Wuhan 430074, China

7 Research Institute of Huazhong University of Science and Technology in Shenzhen, Shenzhen 518057, P. R. China

[†] These authors contributed equally to this work.

*Correspondence authors: Tianyou Zhai (zhaity@hust.edu.cn), Yuanping Yi (ypyi@iccas.ac.cn), Yongli Yan (ylyan@iccas.ac.cn)

Methods

Materials

The C_{60} (99.9%) was purchased from Shanghai Macklin Biochemical Technology Co., Ltd. α-P_4S_3 powders were synthesized with a solid-state reaction. The red P (Alfa 99.9%) and S powders (Alfa Aesar 99.5%) were mixed in molar ratio, ground, and then sealed in a quartz tube with a vacuum condition of about 10^{-5} Torr. The tube was slowly heated up to 350 ℃, kept at this temperature for 48 h, and then cooled to room temperature within 48 h. The obtained product was further purified by recrystallization from excess carbon disulfide under an atmosphere of argon. The toluene (≥ 99.5%) solvent was purchased from Sinopharm Chemical Reagent Co., Ltd. β-P_4S_3 powders were obtained by heating the α-P_4S_3 powders upon 46 ℃ to have a phase transition. C_{60} nanoflakes were prepared by a drop-casting process. 3 mg C_{60} powder was added in 3 ml of tetrachloromethane (Kermel IR) and the solution was sonicated for 5 min. 2 μl of the solution was dropped onto a silicon oxide wafer, and 2D C_{60} with hexagonal shapes were obtained after the solvent fully volatilized. The single $C_{60}\cdot 2P_4S_3$ cocrystals were obtained by the slow evaporation of the toluene solution. In a typical experiment, 7 mg C_{60} and 4 mg α-P_4S_3 were dissolved in 20 ml toluene in a 50 mL beaker at room temperature. The beaker was kept in the dark and covered by a sealing film with small holes, and the solvent evaporated slowly.

Characterizations

Morphology and crystal structure characterizations. The morphology and thickness of $C_{60}\cdot P_4S_3$ nanoflakes were characterized with the optical microscope (BX53M, OLYMPUS), and AFM (Dimension Icon, Bruker). The XRD pattern was measured by a Bruker D2 Phaser X-ray diffractometer. TEM images were obtained by a field emission TEM (Tecnai G2 F30, FEI). SCXRD was performed at room temperature by a Bruker D8venture diffractometer equipped with Cu Kα radiation and the crystal structure was solved and refined using the APEX5 program. The binding energy was characterized by a Kratos AXIS-SUPRA+ X-ray photoelectron spectrometer.

Optical spectra characterizations. Absorption spectra were obtained on a MStarter ABS microscopic absorption spectrometer. Raman spectroscopy, PL spectra, and the fluorescence lifetime were obtained on a confocal Raman spectroscopy (Alpha 300RS+, WITec). A 375 nm pulsed supercontinuum laser is used to measure time-resolved PL spectra at a 4 MHz repetition rate. The polarization-resolved PL spectra were collected with this confocal Raman spectroscopy and the polarizer was rotated 180 ° with a step of 10 ° to collect polarized PL spectra in different orientations. Temperature-dependent PL measurements were conducted using an Attocube attoDry2100 closed-cycle cryogenic microscope. Due to the micron size of the nanoflakes, the PLQY of 2D C_{60} P_4S_3 was measured by the comparative method. 8-Hydroxyquinoline aluminum salt (AIq_3) film was regarded as the reference sample. It was prepared by spin-casting an Alq_3-doped polymethylmethacrylate (PMMA) film onto a glass slide. The precursor solution was prepared by mixing Alq_3 (8 mg/mL, 1 mL, dissolved in dichloromethane) with PMMA (100 mg/mL, 1 mL, dissolved in chlorobenzene). Then, 200 μL of the precursor solution was spin-coated onto a 1 × 1-inch glass substrate at 3000 rpm for 30 seconds. The PLQY of the Alq_3 reference sample was calibrated to be 32.2% under 405 nm excitation using a Hamamatsu C9920-02 PLQY measurement system with an integrating sphere. We measured the integrated PL intensity (I_F, I_R) and absorption value (A_F, A_R) of the C_{60} P_4S_3 nanoflake and the reference sample AIq_3 film, respectively. The PLQY can be determined as:

$$PLQY = Q_R \frac{I_F}{I_R} \frac{A_R}{A_F}$$

The high-pressure technique was performed in a screw-pressure-type Diamond Anvil Cell (DAC) made of nonmagnetic Cu-Be alloy. The culets of the diamond anvils are 300 μm in diameter, which can provide a high pressure of up to 50.0 GPa. A pre-drilled hole (260 μm in diameter) at the center area (pre-indented from 250 to 40 μm in thickness) of a T301 stainless-steel gasket was used as the sample chamber. Ruby micro balls were loaded into the sample chamber to calibrate the precise value of applied pressure with room-temperature R1 ruby photoluminescence. The 2D

$C_{60}\cdot 2P_4S_3$ crystals on the diamond culet of DAC were prepared by a standard dry-transfer process, where the samples were first transferred from the silicon oxide wafer onto the onto the polydimethylsiloxane (PDMS) and then from PDMS to diamond surface in an N_2 glovebox.

Optical waveguide characterizations. The prepared thick $C_{60}\cdot 2P_4S_3$ crystals on the MgF_2 substrate were used for the optical waveguide test. The microarea PL measurements were performed on a home-made confocal micro-PL system that could locally excite the crystals with a 405 nm laser beam focused with an objective lens (Nikon CFLU Plan, 50×). The optical waveguides were detected using a vertically excited mode, i.e., the excitation direction was perpendicular to the propagation direction. The molecular crystals were irradiated at different locations with a constant laser intensity. The emission of the crystal end was selectively collected from the tip using a confocal microscopy setup with a 1 mm pinhole. The light was subsequently coupled to a grating spectrometer (Acton SP2300i) with a matched thermally electrically cooled charge-coupled device (Princeton Instruments, ProEm: 1600 × 200B).

Theoretical calculations

Electronic structure calculation of isolated molecule: all electronic structure calculations were carried out using Gaussian 16 package.[1] Geometrical structure optimization was performed before all isolated molecule calculation at the level of PBE0/6-31G(d) with DFT-D3(BJ) dispersion correction. No imaginary frequency mode was found in the following frequency calculation. Hirshfeld surface of clusters was generated by Multiwfn software.[2-4] The Hirshfeld surface is constructed as the 0.5 isosurface of the Hirshfeld electronic sharing function (ρ), originally defined by Hirshfeld, F.L.[5] The fingerprint diagrams represent a quantitative analysis derived from the Hirshfeld surface. In practice, the program samples points across the entire Hirshfeld surface and calculates two parameters for each point: d_i, the distance to the nearest atom of the core molecule, and d_e, the distance to the nearest atom of the surrounding molecules in the crystal structure. The resulting 2D plots, known as

fingerprint diagrams, illustrate the distribution of intermolecular contacts. The color scale indicates the density of sampled points. A local contact fingerprint diagram further filters these points by specified interaction types, thereby correlating particular elements with weak intermolecular interactions.

Electronic structure calculation of period system: all calculations for the period system were carried out using CRYSTAL17 package.[6] Geometrical structure of $C_{60}\cdot 2P_4S_3$ and β-P_4S_3 crystals were obtained from experimental result mentioned in previous statement. And the crystal structure of C_{60} was downloaded from Materials Project website.[7, 8] SCF calculation performed at the level of PBE0/pob-DZVP-rev2 with DFT-D3 dispersion correction. All subsequent results are derived from the SCF wavefunctions.

Vibrationally-resolved electronic spectra simulation: frequency calculation was performed at the level of PBE0/6-31G(d) with DFT-D3(BJ) dispersion correction. Gaussian 16 package was used during calculation.[1] Time-independent Vibrationally-resolved electronic spectra simulation was carried out by FCClasses 3 package.[9] Herzberg-Teller effect was considered during the calculation. Gaussian function with 0.036 eV HWHM was used to broadening the spectra.

Supplementary figures and tables

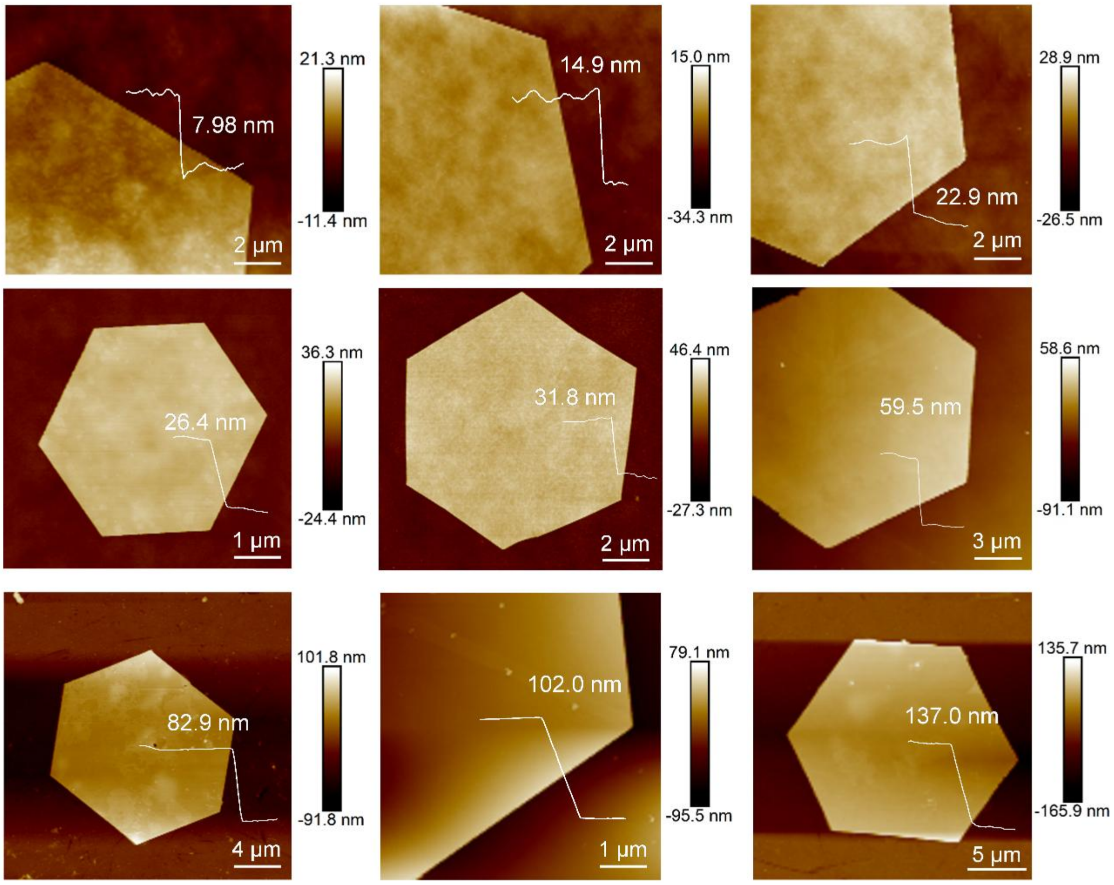

Figure S1. AFM images and height profiles of 2D $C_{60} \cdot 2P_4S_3$ nanoflakes prepared by the drop-casting method.

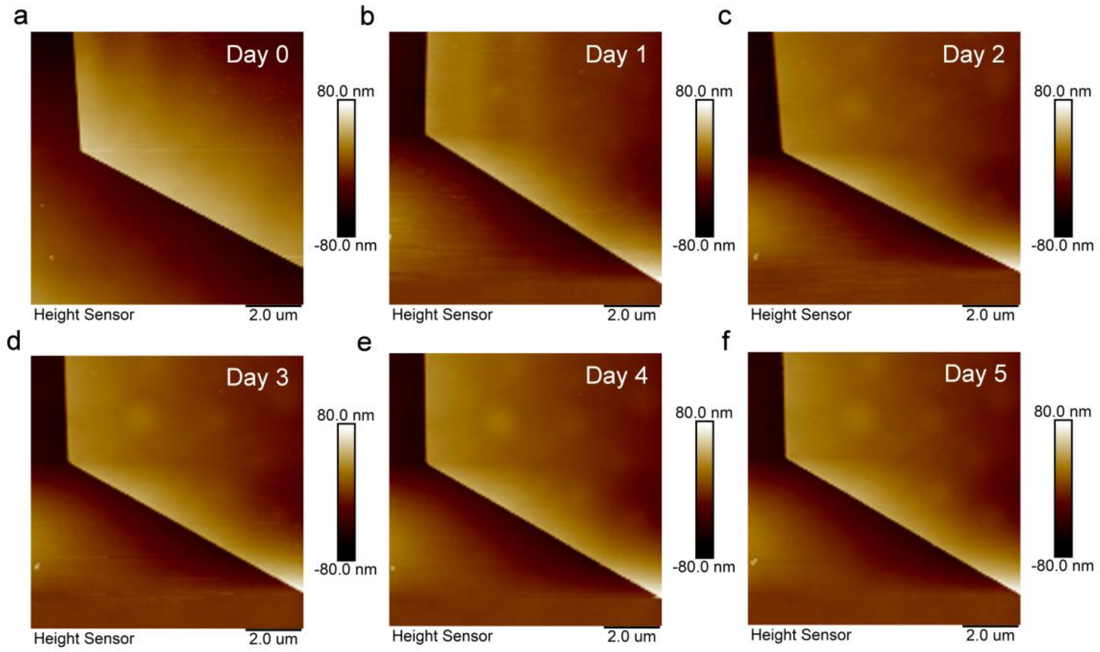

Figure S2. AFM images of $C_{60}\cdot 2P_4S_3$ nanoflakes stored in a environmental chamber (25℃, 100% RH) over five days: (a) Day 0, (b) Day 1, (c) Day 2, (d) Day 3, (e) Day 4, and (f) Day 5.

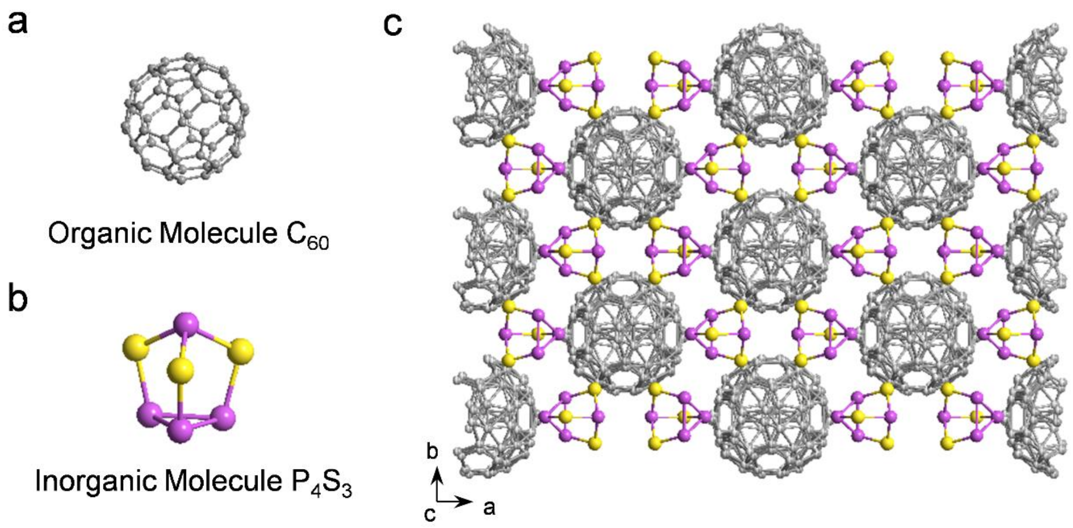

Figure S3. Schematics of crystal structure, (a) C_{60} molecule, (b) P_4S_3 molecule, (c) $C_{60} \cdot 2P_4S_3$ cocrystal along the c-axis direction.

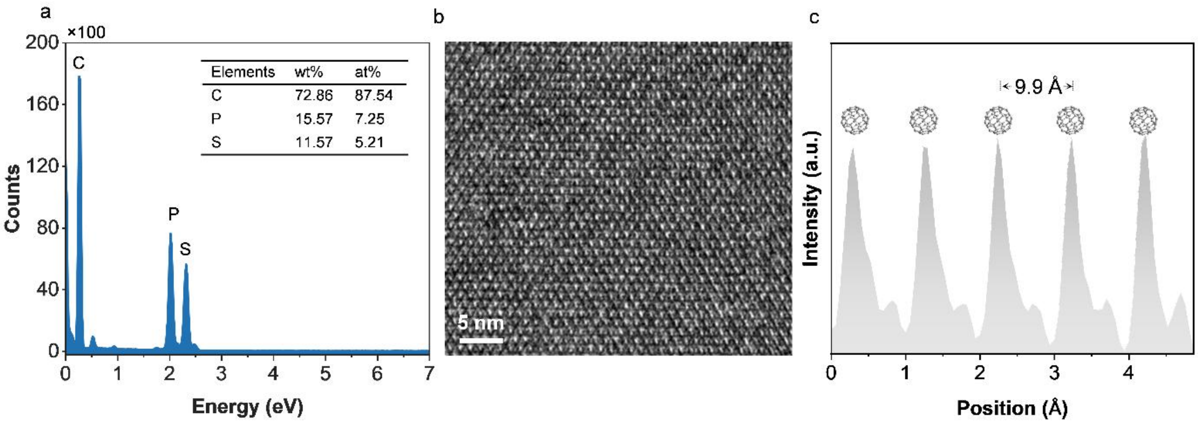

Figure S4. TEM characterization of 2D C_{60} ·$2P_4S_3$ cocrystal. (a) EDX spectrum. The inset table shows the weight and atom percentage of C, P and S element in the cocrystal. (b) HRTEM image. (c) Intensity line profile along the yellow box in Figure 1f.

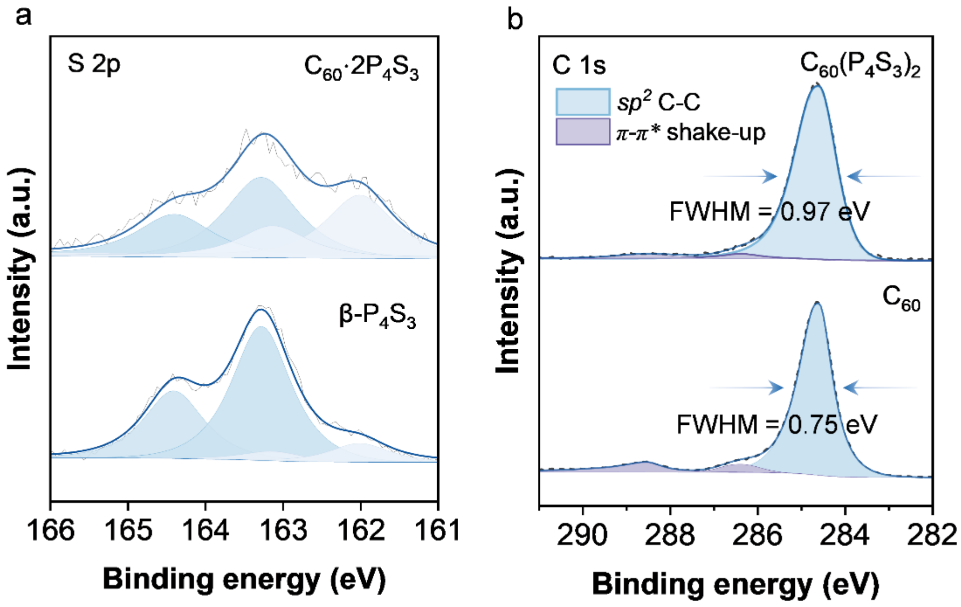

Figure S5. XPS spectra for the as-grown $C_{60}\cdot 2P_4S_3$ nanoflakes, β-P_4S_3 particles and C_{60} particles. (a) S 2p. (b) C 1s.

For comparison, β-P_4S_3 was selected as the reference due to its disordered plastic phase suppressing intermolecular interactions and better approximating isolated single-molecule behavior, while the α-phase reportedly has strong intermolecular contact and hence the electron density distortions.[10, 11] Compared with pristine C_{60}, the C 1s spectrum of $C_{60}\cdot 2P_4S_3$ shows unchanged peak positions for the sp ^{2}C-C bond and the π-π shakeup features at 284.6 eV, 286.38 eV, and 288.55 eV, respectively. The full width at half maximum (FWHM) of the sp ^{2}C-C bond peak increases from 0.75 eV in C_{60} to 0.97 eV in $C_{60}\cdot 2P_4S_3$, corresponding to a broadening of 0.22 eV). This peak broadening indicates that the strong intermolecular C-P contacts in the cocrystal modify the local electronic environment of the C atoms, further supporting the presence of strong intermolecular interactions.[12, 13]

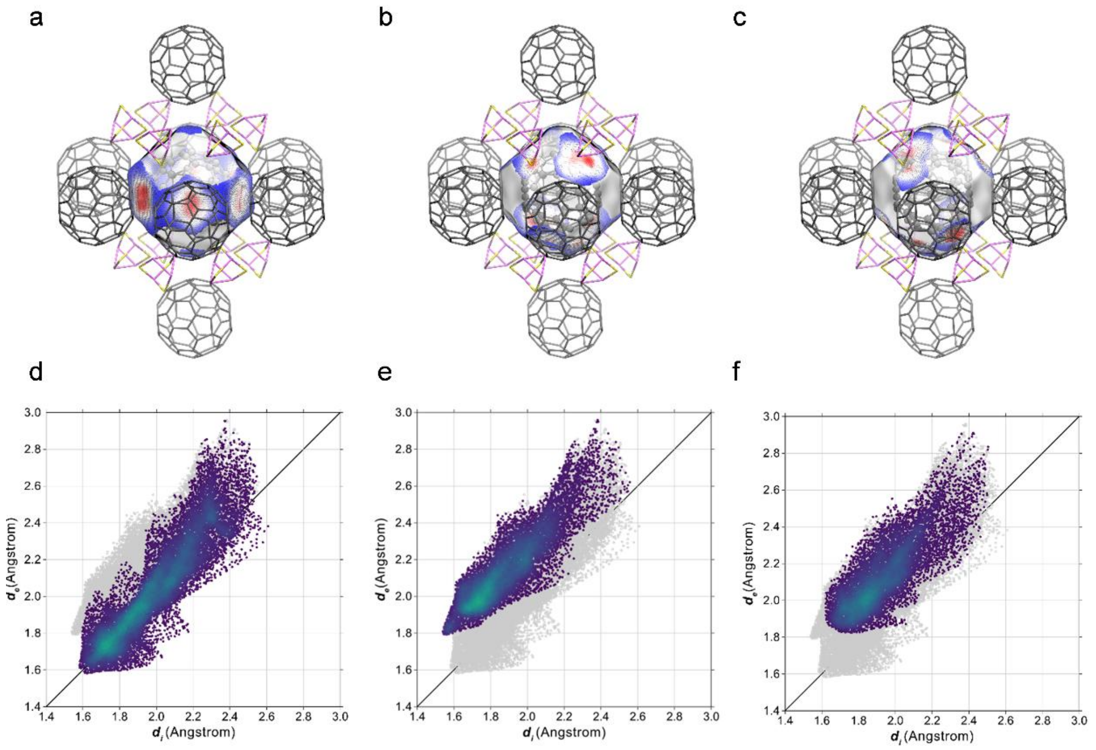

Figure S6. Local contact analysis of Hirshfeld surface and corresponding fingerprint plot of (a, d) C, (b, e) P, (c, f) S in C_{60} ·$2P_4S_3$.

The local contact analysis filters the sample points from the entire Hirshfeld surface and fingerprint diagram according to different external atoms, a carbon atom (Figures S5a, d), a phosphorus atom (Figures S5b, e), and a sulfur atom (Figures S5c, f), in contact with the carbon atom inside.

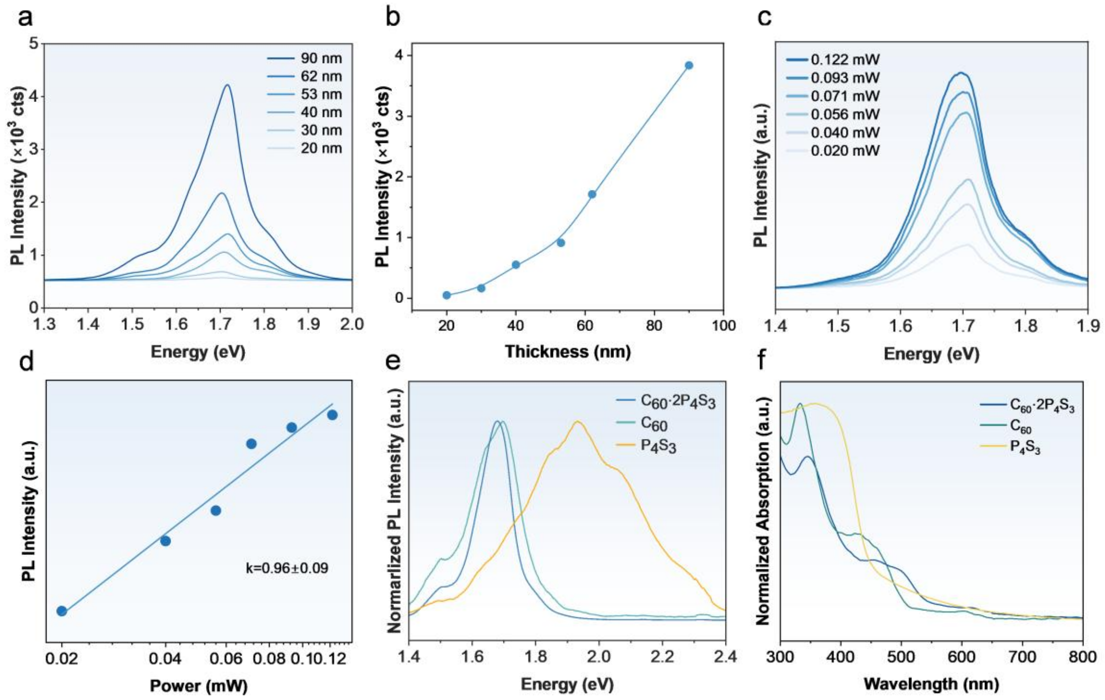

Figure S7. Optical spectra of C_{60} ·2P_4S_3**.** (a) PL spectra of C_{60} ·2P_4S_3 nanoflakes with different thicknesses. (b) The relationship between PL intensity and thickness. (c) PL spectra of C_{60} ·2P_4S_3 with excitation power from 0.020 to 0.122 mW. (d) Integrated PL intensity versus excitation density. (e) Normalized PL spectra of the as-grown C_{60} ·2P_4S_3, C_{60} and P_4S_3 particles under the same measurement conditions (532 nm excitation, 0.1 mW power, 1s integration time). (f) Absorption spectra of C_{60} ·2P_4S_3, C_{60} and P_4S_3 crystals.

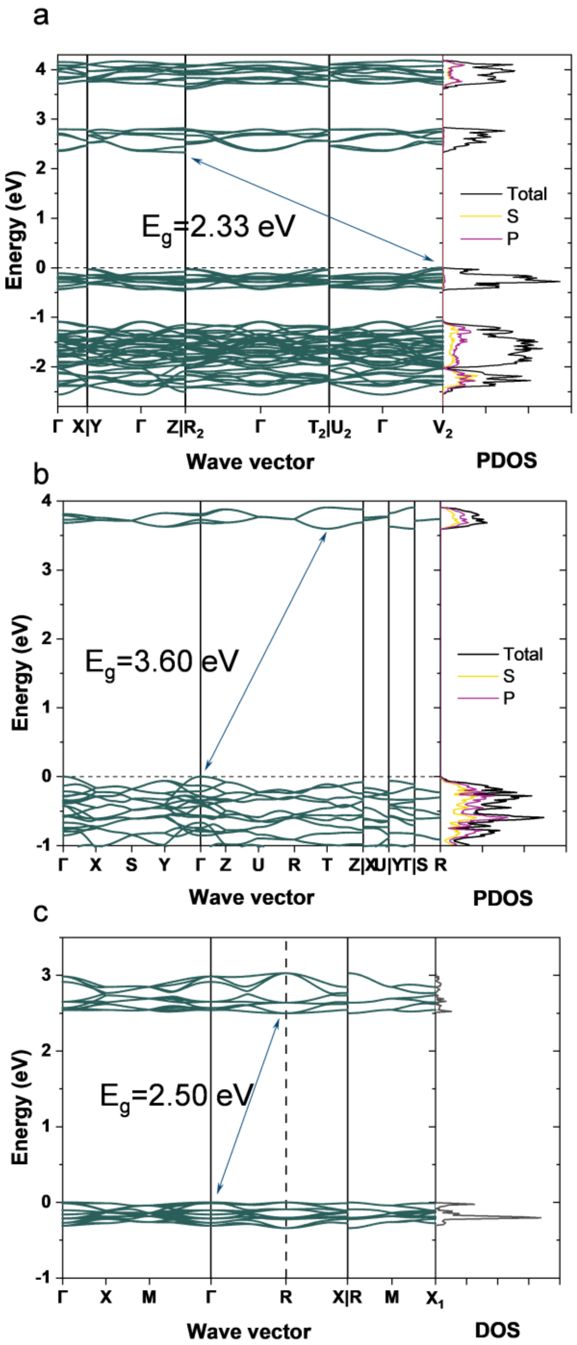

Figure S8. Calculated band diagram and DOS of (a) C_{60} $2P_4S_3$, (b) P_4S_3, (c) C_{60}.

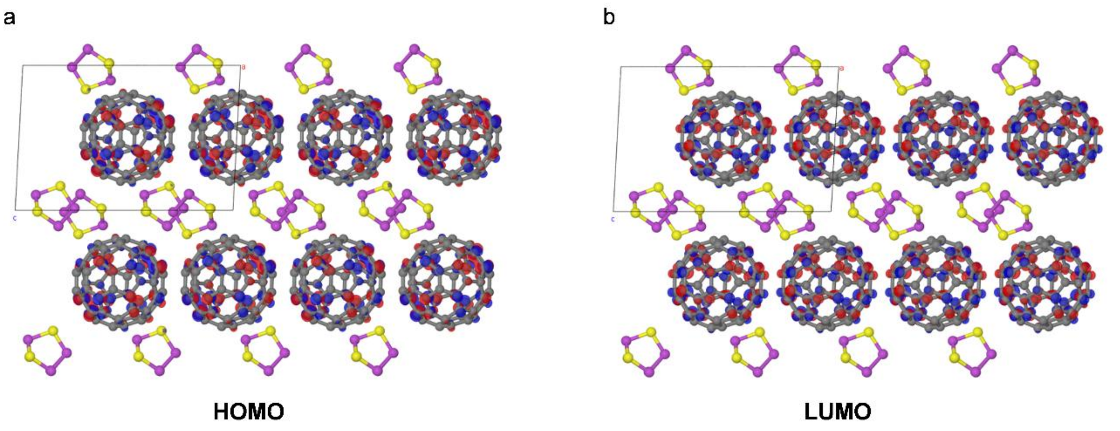

Figure S9. Calculated (a) HOMO and (b) LUMO orbitals of $C_{60}\cdot 2P_4S_3$. The HOMO and LUMO orbitals are only localized on the C_{60} molecules.

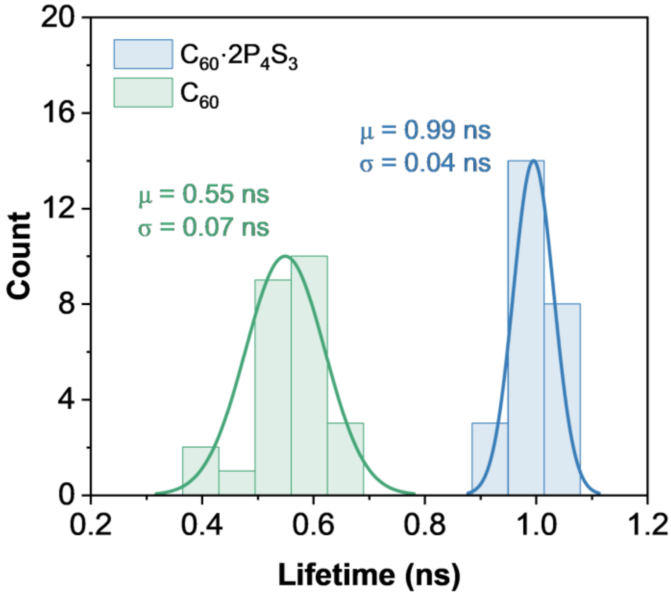

Figure S10. Statistical analysis of PL lifetimes for C_{60} ·$2P_4S_3$ and C_{60} nanoflakes.

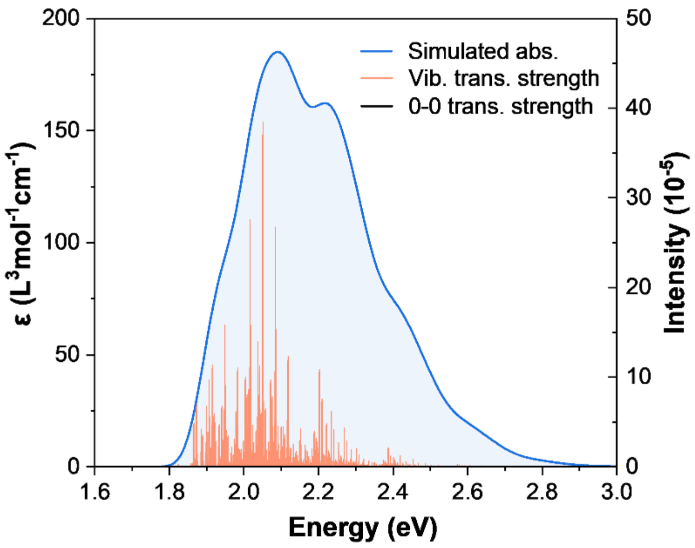

Figure S11. Simulated one photon absorption spectrum.

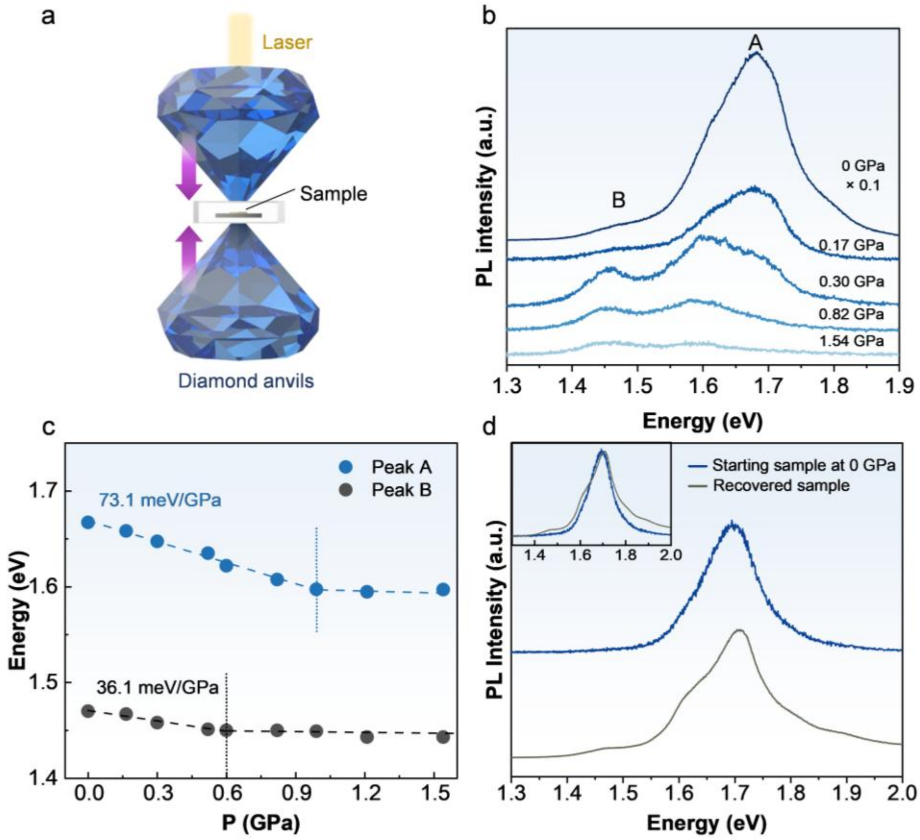

Figure S12. Pressure-dependent PL spectra of 2D $C_{60}\cdot 2P_4S_3$ cocrystals. (a) Illustration of the experimental setup for in-situ high-pressure PL characterization. (b) PL spectra of 2D $C_{60}\cdot 2P_4S_3$ cocrystals under different pressures. (c) The emission energy of the cocrystal as a function of pressure. (d) PL spectra of $C_{60}\cdot 2P_4S_3$ cocrystals before and after applying the pressure of 1.5 GPa.

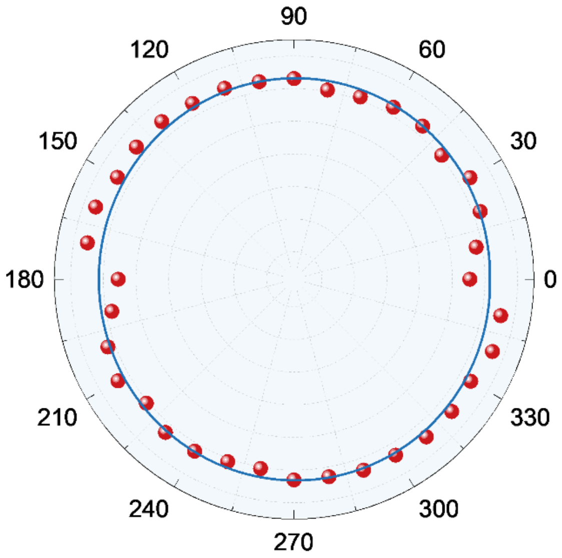

Figure S13. Polar plot of the PL intensity of C_{60} nanoflake.

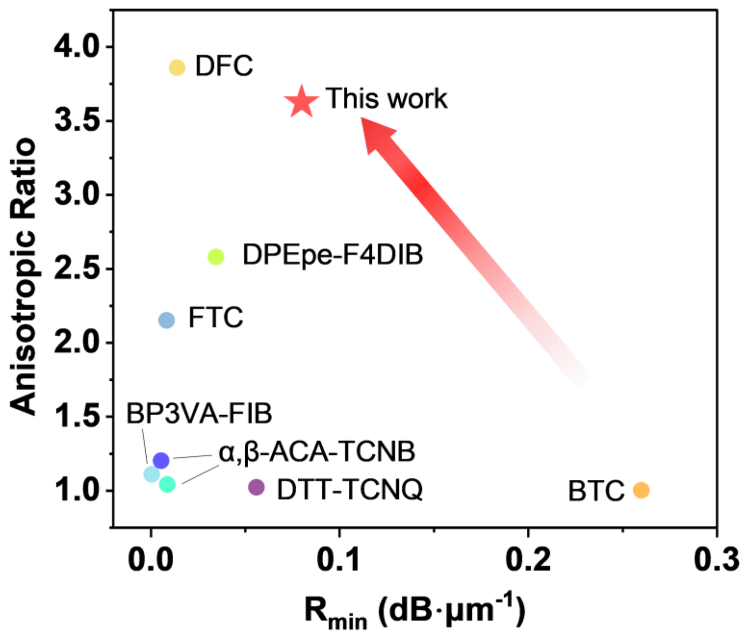

Figure S14. Optical waveguide performance of representative organic cocrystals [14-20]. The x axis corresponds to the minimum propagation loss coefficient and the y axis corresponds to the anisotropic ratio.

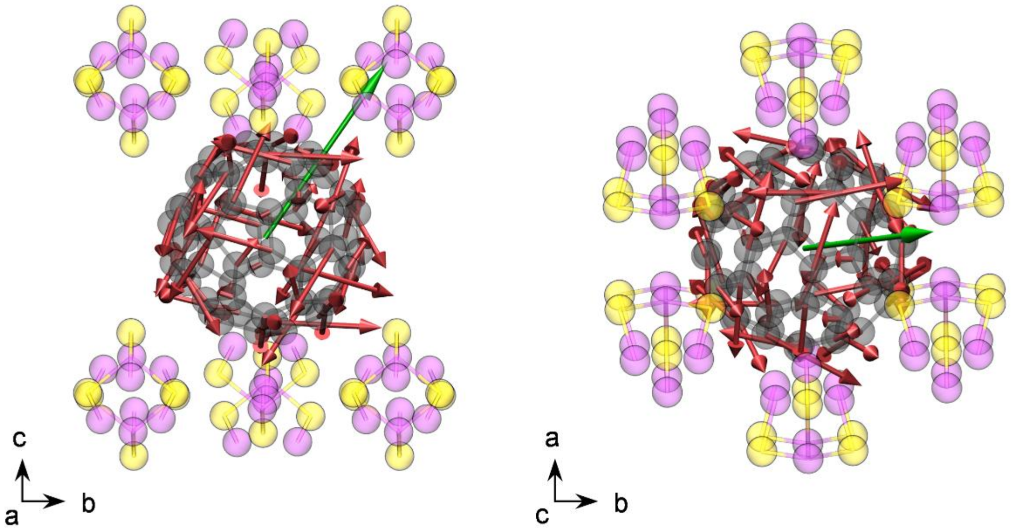

Figure S15. Schematic of derivatives of transition dipole moment from mode 157.

Table S1. Crystal data and structure refinement for the $C_{60}\cdot 2P_4S_3$ cocrystal.

Empirical formula	$C_{60}P_8S_6$
Formula weight	1160.72
Temperature/K	300
Crystal system	monoclinic
Space group	C2/m
a/Å	17.4098(6)
b/Å	9.8283(3)
c/Å	11.6240(4)
α/°	90
β/°	92.832(3)
γ/°	90
Volume/$Å^3$	1986.54(11)
Z	2
ρ_{calc}g/cm^3	1.940
μ/mm^{-1}	6.670
F(000)	1152.0
Crystal size/mm^3	0.2 × 0.1 × 0.1
Radiation	Cu Kα (λ = 1.54178)
2θ range for data collection/ °	7.614 to 133.742
Index ranges	$-20 \le h \le 19$, $-11 \le k \le 11$, $-13 \le l \le 13$
Reflections collected	9612
Independent reflections	1843 [Rint = 0.0365, Rsigma = 0.0224]
Absorption correction	Multi-scan
Data/restraints/parameters	1843/13097/224
Goodness-of-fit on F^2	1.096
Final R indexes [I>=2σ (I)]	R_1= 0.0924, wR_2= 0.2656
Final R indexes [all data]	R_1= 0.0947, wR_2= 0.2669
Largest diff. peak/hole / e $Å^{-3}$	1.33/-0.67

Table S2. XPS data of the 2D C_{60} ·$2P_4S_3$ cocrystal.

**Element**	**Orbital**	**Binding Energy/eV**	
		**In $C_{60}(P_4S_3)_2$**	**In β-P_4S_3**
P	$2p_{1/2}(1)$	134.54	135.67
	$2p_{3/2}(1)$	133.70	134.83
	$2p_{1/2}(2)$	131.54	132.06
	$2p_{3/2}(2)$	130.70	131.22
S	$2p_{1/2}(1)$	164.4	164.41
	$2p_{3/2}(1)$	163.28	163.28
	$2p_{1/2}(2)$	163.13	163.13
	$2p_{3/2}(2)$	162.01	162.01

Table S3. Summary of PLQY values of fullerene-based luminescent materials reported in the literature.

	**PLQY**	**References**
C_{60} molecule	0.01%	[21]
C_{60} crystal	0.07%	[22]
C_{60} m-xylene	0.2%	[23]
Rubrene/C_{60}	0.075%	[24]
C_{60}@Nanographene	1%	[25]
This work	13.24%	

Table S4. Displacement of electronic vibration bands with respect to the source and possible attribution of these bands based on Raman frequencies for 2D $C_{60} \cdot 2P_4S_3$.

E (eV)	λ (nm)	σ (cm^{-1})	Δσ (cm^{-1})	Assignment
1.776	698.1982	14322.58		
1.712	724.2991	13806.45	516.1284	530 (unknown)
1.681	737.6562	13556.45	766.1284	772 ^{4}Hg
1.657	748.3404	13362.9	959.6768	1100 ^{5}Hg
1.625	763.0769	13104.84	1217.741	1248 ^{6}Hg
1.599	775.4847	12895.16	1427.419	1422 ^{7}Hg
1.536	807.2917	12387.1	1935.483	1427+516

Table S5. Calculated major vibronic transition assignments of C_{60} $2P_4S_3$ with intensity higher than 10^{-4}.

Assignment	Energy(eV)	Intensity (10^{-5})
$\vert S_1>\rightarrow\vert S_0;157^{1}>$	1.634	15.855
$\vert S_1>\rightarrow\vert S_0;162^{1}>$	1.633	12.142
$\vert S_1>\rightarrow\vert S_0;173^{1}>$	1.617	19.792
$\vert S_1>\rightarrow\vert S_0;175^{1}>$	1.617	11.159
$\vert S_1>\rightarrow\vert S_0;173^{1},1^{1}>$	1.616	11.670
$\vert S_1>\rightarrow\vert S_0;156^{1},2^{1}>$	1.633	12.533
$\vert S_1>\rightarrow\vert S_0;157^{1},2^{1}>$	1.633	21.627
$\vert S_1>\rightarrow\vert S_0;157^{1},2^{2}>$	1.632	17.097
$\vert S_1>\rightarrow\vert S_0;157^{1},2^{3}>$	1.630	10.145
$\vert S_1>\rightarrow\vert S_0;162^{1},2^{1}>$	1.632	16.562
$\vert S_1>\rightarrow\vert S_0;162^{1},2^{2}>$	1.631	13.093
$\vert S_1>\rightarrow\vert S_0;172^{1},2^{1}>$	1.615	11.894
$\vert S_1>\rightarrow\vert S_0;173^{1},2^{1}>$	1.615	26.996
$\vert S_1>\rightarrow\vert S_0;173^{1},2^{2}>$	1.614	21.341
$\vert S_1>\rightarrow\vert S_0;173^{1},2^{3}>$	1.613	12.662
$\vert S_1>\rightarrow\vert S_0;175^{1},2^{1}>$	1.615	15.220
$\vert S_1>\rightarrow\vert S_0;175^{1},2^{2}>$	1.614	12.032
$\vert S_1>\rightarrow\vert S_0;157^{1},11^{1}>$	1.600	14.272
$\vert S_1>\rightarrow\vert S_0;162^{1},11^{1}>$	1.599	10.929
$\vert S_1>\rightarrow\vert S_0;173^{1},11^{1}>$	1.583	17.803
$\vert S_1>\rightarrow\vert S_0;175^{1},11^{1}>$	1.582	10.037

Table S6. Calculated derivatives of transition dipole moment weighted by $1/\sqrt{\nu}$ from high-frequency modes (projected to cell axis).

Mode	Frequency $\underline{\nu}$ (cm^{-1})	c (a.u.)	a (a.u.)	b (a.u.)
157	1500.77	0.0656	0.0059	0.0455
173	1643.26	0.0770	-0.0326	0.0282

References

1. Frisch M J, Trucks G W, Schlegel H B , et al. Gaussian, 2016, Inc., Wallingford CT.
2. Lu T, Chen F. Quantitative analysis of molecular surface based on improved Marching Tetrahedra algorithm. *J Mol Graphics Modell*. 2012; **38**: 314-323.
3. Lu T, Chen F. Multiwfn: A multifunctional wavefunction analyzer. *J Comput Chem*. 2012; **33**: 580-592.
4. Lu T. A comprehensive electron wavefunction analysis toolbox for chemists, Multiwfn. *J Chem Phys*. 2024; **161**: 082503.
5. Hirshfeld FL. Bonded-atom fragments for describing molecular charge densities. *Theor Chim Acta*. 1977; **44**: 129-138.
6. Dovesi R, Erba A, Orlando R *et al.* WIREs Comput. *Mol Sci*. 2018; **8**: e1360.
7. Dorset DL, McCourt MP. Disorder and the molecular packing of C_{60} buckminsterfullerene: a direct electron-crystallographic analysis. *Acta Crystallogr, Sect A: Found Crystallogr*. 1994; **50**: 344-351.
8. Jain A, Ong SP, Hautier G *et al.* Commentary: The Materials Project: A materials genome approach to accelerating materials innovation. *APL Mater*. 2013; **1**: 011002.
9. Cerezo J, Santoro F. FCclasses3: Vibrationally-resolved spectra simulated at the edge of the harmonic approximation. *J Comput Chem*. 2023; **44**: 626-643.
10. Chattopadhyay T, Gmelin E, Schnering H. Thermal properties of P_4S_3 in the crystalline and in the plastic state. *J Phys Chem Solids*. 1982; **43**: 925-932.
11. Feng X, Peng X, Peng B *et al.* Effect of strong intermolecular interaction in 2D inorganic molecular crystals. *J Am Chem Soc*. 2021; **143**: 20192-20201.
12. Onoe J, Nakao A, Takeuchi K. XPS study of a photopolymerized C_{60} film. *Phys Rev B*. 1997; **551**: 65617-10056.
13. Hou L, Cui X, Guan B *et al.* Synthesis of a monolayer fullerene network. *Nature*. 2022; **606**: 507-510.
14. Du S, Ma S, Xu B *et al.* Optical waveguide and photoluminescent polarization in organic cocrystal polymorphs. *J Phys Chem Lett*. 2021; **12**: 9233-9238.
15. Wang J, Xu S, Li A *et al.* Polymorphism-based luminescence and morphology-dependent optical waveguide properties in 1: 1 charge transfer cocrystals. *Mater Chem Front*. 2021; **5**: 1477-1485.
16. Xu C-F, Liu Y-P, Yu Y *et al.* Two-dimensional optical waveguides at telecom wavelengths based on organic single-crystal microsheets of a charge transfer complex. *J Phys Chem Lett*. 2023; **14**: 3047-3056.
17. Zhu W, Zheng R, Fu X *et al.* Revealing the charge-transfer interactions in self-assembled organic cocrystals: two-dimensional photonic applications. *Angew Chem Int Ed*. 2015; **54**: 6785-6789.
18. Ye X, Liu Y, Guo Q *et al.* 1D versus 2D cocrystals growth via microspacing in-air sublimation. *Nat Commun*. 2019; **10**: 761.
19. Zhuo MP, Tao YC, Wang XD *et al.* 2D Organic Photonics: An Asymmetric Optical Waveguide in Self-Assembled Halogen-Bonded Cocrystals. *Angew Chem Int Ed*. 2018; **130**: 11470-11474.
20. Liu Y, Hu H, Xu L *et al.* Orientation-Controlled 2D Anisotropic and Isotropic Photon Transport in Co-crystal Polymorph Microplates. *Angew Chem Int Ed*. 2020; **59**: 4456-4463.
21. Catalan J, Elguero J. Fluorescence of fullerenes (C_{60} and C_{70}). *J Am Chem Soc*. 1993; **115**: 9249-9252.
22. Guss W, Feldmann J, Göbel EO *et al.* Fluorescence from X traps in C_{60} single crystals. *Phys Rev Lett*. 1994; **72**: 2644-2647.

23. Wang L, Liu B, Yu S *et al.* Highly Enhanced Luminescence from Single-Crystalline C_{60}·1m-xylene Nanorods. *Chem Mater*. 2006; **18**: 4190-4194.
24. Ullah M, Yambem SD, Moore EG *et al.* Singlet Fission and Triplet Exciton Dynamics in Rubrene/Fullerene Heterojunctions: Implications for Electroluminescence. *Adv Electron Mater*. 2015; **1**: 1500229.
25. Chai L, Ju Y-Y, Xing J-F *et al.* Nanographene Metallaprisms: Structure, Stimulated Transformation, and Emission Enhancement. *Angew Chem Int Ed*. 2022; **61**: e202210268.
